# Chunking, Attraction, Repulsion, and Ensemble Effects Are Ubiquitous in Visual Working Memory

**DOI:** 10.1162/OPMI.a.312

**Published:** 2026-02-15

**Authors:** Chaipat Chunharas, Timothy F. Brady

**Affiliations:** Cognitive Clinical & Computational Neuroscience Center of Excellence, Department of Internal Medicine, Faculty of Medicine, Chulalongkorn University; Chula Neuroscience Center, King Chulalongkorn Memorial Hospital, Thai Red Cross Society, Bangkok, Thailand; Department of Psychology, University of California, San Diego, La Jolla, CA, USA

**Keywords:** working memory capacity, chunking, ensemble perception, visual working memory

## Abstract

What happens when there are many different objects that must be maintained simultaneously in visual working memory? Prominent models focus largely on individual objects in describing memory limits: e.g., claims that we can remember only a fixed number of items, or that a single resource limit describes the cost of holding additional items. While some acknowledge interactions between items, these interactions are not given a prominent role in most models. Here we show that instead of items being represented independently, the visual display is always compressed by utilizing clusters of items and the gist of the display. We reanalyze data from 11 previously available datasets (comprising 137,986 trials in total). We find strong evidence for non-independent representations, including chunking and use of the ‘gist’ of the display, which is present in nearly every study at every set size. We then present a model for understanding this chunking and the resulting systematic biases (e.g., items attracting and repelling one another) based on psychophysical similarity. Overall, this work provides strong evidence that in order to understand visual working memory, we need to consider how our memory system takes advantage of the relationship between items. This is in contrast to the way the majority of the field studies visual working memory and suggests a major paradigm shift is required to think of memory in terms of clusters, chunks, and gist rather than in terms of independent items.

## INTRODUCTION

Visual working memory is the system we use to hold visual information actively in mind so we can manipulate it, or use it to guide our attention or eye movements (e.g., Brady et al., 2011; Hollingworth et al., 2008). This system is strongly capacity limited: When people are asked to remember more than a few items in visual working memory, performance suffers. Most prominent models of such capacity limits focus on limits at the level of individual items. For example, many prominent models claim performance suffers because a subset of items is unrepresented (e.g., Zhang & Luck, 2008); others claim a subset of items is represented extremely poorly, and this is increasingly common as set size increases (van den Berg et al., 2012); others claim the signal-to-noise of all items drops when more must be maintained (e.g., Schurgin et al., 2020), perhaps because of neural limits on how much information can be represented effectively (e.g., Bays, 2015). These models all have in common that they treat the display as fully individuated: as though ‘slots’ or ‘resources’ are allocated item-by-item, and memory for each item is relatively independent of the other items. While some models allow that interference between items might be a relevant consideration (e.g., Oberauer & Lin, 2017; Swan & Wyble, 2014), these models still treat individuated items as the relevant unit of representation over which interference occurs.

A large amount of work has shown, in particular in structured displays where items are not randomly chosen, that working memory does not consist solely of independent memory for individual items (e.g., Brady et al., 2011). For example, existing work has shown that in displays that are not fully random—instead generated based on systematic interactions between a pair of items (Bae & Luck, 2017; Chunharas et al., 2019; Golomb, 2015) or some rules that create ‘clusters’ in the features of many items—people rely on summary statistics of the display, like the mean of the entire display and the mean of the relevant subset or cluster of the display (e.g., Brady & Alvarez, 2011; Lew & Vul, 2015; Orhan & Jacobs, 2013; Orhan et al., 2014; Son et al., 2020; Utochkin & Brady, 2020). This results in biases in memory toward the mean of a cluster (e.g., Brady & Alvarez, 2011; Lew & Vul, 2015; Orhan et al., 2014) and improved precision for items in clusters (Orhan et al., 2014; Sims et al., 2012; Utochkin & Brady, 2020). Such non-item-based representations can be quite complex, reflecting much more than just summary statistics, taking into account spatial distributions (as in texture representations; Brady & Alvarez, 2015b; Brady & Tenenbaum, 2013; Boduroglu & Yildirim, 2020; Schurgin & Brady, 2019); or chunk or similarity structure (Nassar et al., 2018; Son et al., 2020), and can reflect the and variance of features, not just averages (e.g., Utochkin & Brady, 2020). In situations where stimuli are reliably mutually informative with each other across trials, consistently forming clusters or chunks, this work clearly shows that people take advantage of this to encode the displays more efficiently. The relationship between distinct objects in the same scene in the real world is clearly non-random—e.g., items and features show strong co-occurrence regularities in real images (e.g., Barlow, 1989; Field, 1987; Greene, 2013)—and object features are strongly non-random in realistic objects, providing memory advantages (e.g., Hedayati et al., 2022; O’Donnell et al., 2018; Stoermer & Brady, 2021). Thus, this work strongly suggests that real-world working memory encoding is not based on independent representations of items, and that more structured memory representations must be considered in working memory models.

However, there is a rich tradition in visual working memory research, and working memory research more broadly, of attempting to understand a putative ‘core’ working memory capacity that is distinct from how working memory is used in ecological settings where such regularities and prior knowledge is present. This line of thought is exemplified by Cowan (2001)’s influential paper which treats the ability to extract relations among items and thus form ‘chunks’ as a nuisance factor that must be removed with careful experimental design in order to assess the *true number* of independent items that can be represented. To attempt to eliminate such chunking, some researchers believe that to tap into the pure working memory capacity, we must use very short encoding times (e.g., Lin & Luck, 2012; Quirk et al., 2020); meaningless stimuli that repeat trial-to-trial (e.g., Lin & Luck, 2012); prevent verbal re-encoding (e.g., Levy, 1971); eliminate all possible spatial regularities that could allow for chunking and eliminate prior knowledge that can be used to inform the items in the display (e.g., Cowan, 2001). Under these conditions, the idea is, items are represented independently and there are fixed limits on how many can be represented (e.g., Awh et al., 2007; Fukuda et al., 2010; Luck & Vogel, 2013). This view is implicit in nearly all of the computational approaches that are based on measures of resources or slots devoted to independent items (e.g., Adam et al., 2017; Schneegans et al., 2020; Schurgin et al., 2020). Do such conditions truly exist? That is, is there any experiment where people do not use the relations between items to compress the display and genuinely store independent items? In the current work, we test the independence of items in data that we believe provides the strongest possible test of whether methodological choices are responsible for non-independent and structured representations in working memory: existing data that was used to argue for purely independent item-based representations.

Here we provide a strong demonstration that this strictly item-based approach to performance is inaccurate across every public visual working memory dataset we analyzed (11 in total). Rather than thinking about each item independently, we show that when there is more than 1 item to remember, people encode items relative to each other and take advantage of the structure of the display to form clusters and extract gist-based summaries. To make this point as forcefully as possible, we show this is true even in conditions explicitly designed to make items as separate as possible and prevent any complex encoding strategies, and that have been used to argue for item-specific representation.

In particular, we focus on reanalysis of data that has been interpreted as proving item-based limits in working memory (Adam et al., 2017), as well as data from a large number of labs that has likewise been interpreted as solely reflecting item-based storage, including in one case a meta-analysis making the explicit claim that no ensemble or clustering effects were found in such data (van den Berg et al., 2014). We argue that our finding of strong inter-item effects in all of these datasets demonstrates that in all cases, the units of memory are not individual items, but rather structured representations which cluster items based on similarity in features (e.g., Brady & Alvarez, 2011, 2015a; Nassar et al., 2018; Son et al., 2020).

In the first part, we reanalyzed one of the richest publicly available working memory datasets provided by Adam et al. (2017) and found strong evidence for clustering, chunking, and non-item-based representations in this data. In particular, people tend to report the colors that are close to the mean color of the display first, and these first responses are systematically attracted toward each other (e.g., reported closer together in color space than the items truly had been). The number of items that are grouped depends on the chunkability of the display (more chunkable leads to more grouping of responses). Examining the last items people report on a given display also reveals they are not random responses. Instead, the later responses (including the last) are systematically repelled from the earlier ones. Thus, rather than encoding items independently as supposed by both Adam et al. (2017) and Schneegans et al. (2020), memoranda are compressed by combining and separating items in relation to each other, using clusters.

In the second section of the paper, we expand the analyses motivated by Adam et al. (2017)’s data to a wide variety of other datasets made publicly available by van den Berg et al. (2014). We extended the idea of Target Confusability Competition (TCC) model that assumes that each target will activate nearby features and developed a TCC-motivated chunking model. The gist of the display is simply a summation of all those psychological-distance functions. By grabbing the “peak” of the summed functions, we can identify the feature (position of the peak on the feature space), the strength (height of the peak) as well as the number of gists (number of peaks). We find the same effects are pervasive: displays that are more chunkable are better remembered at every set size in every experiment; these chunks produce systematic attraction and repulsion effects (i.e., biases where items are systematically reported as more or less similar to other items on the display); and differential effects across different items, depending on how well they are captured by such chunks.

Overall, we show that no memorandum is an island and each response reflects how that item fits into the whole picture. We argue that it is critical to take into account such effects in all models that attempt to understand how performance changes across set sizes and in considering the basic cognitive architecture of working memory.

## EXPERIMENTAL RE-ANALYSIS 1: WHOLE PROBE COLOR REPORT DATA

### Methods

**Figure 1.**
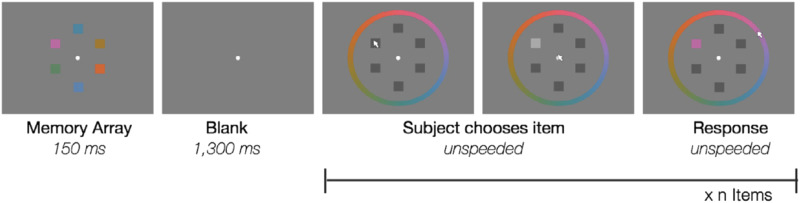
Methods of Adam et al. Experiment 1A. Participants saw 1–6 items and then after a delay reported all items, one at a time, using a continuous color wheel.

We first performed a re-analysis of the freely available data from Adam et al. (2017), Experiment 1A. In their task, they had participants remember 1–6 briefly presented colors (150 ms) over a delay (1000–1300 ms) (Figure 1). After the delay, participants reported each item from the display, one at a time, in whatever order they wished, using a continuous color wheel. This gives as a unit of analysis the angular difference between the response color and memory color for each reported item on each trial. There were 22 participants, and each performed 5 blocks of 99 trials (1 block for each set size).

### Results

#### Accuracy Decreased in Later Responses.

**Figure 2.**
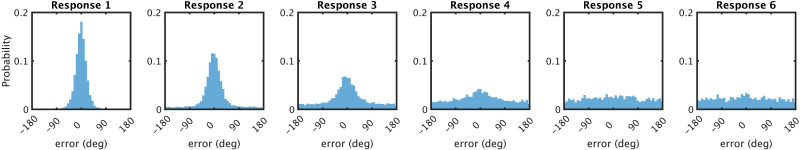
Results of Adam et al. Experiment 1A. The first items people respond to are accurately reported (errors near 0), but later items approximate uniform distributions, suggesting little information is present about these items.

The critical result in Adam et al. (2017) is that accuracy decreased in later responses, with these distributions being more spread out and close to a uniform distribution (Figure 2).

#### Responses Were Not Independent.

We first examined the data for systematic relationships between each response and each memory item. Since in Adam et al.’s stimulus displays, the memoranda were totally independent of each other (e.g., each generated at random with no constraints on their similarity), then if all of the memory items were maintained independently, we should only find systematic relationship when the response is plotted against the target memory item (e.g., response #1 and memory item #1; response #2 and memory item #2 and so on). In these pairings of a response with the item it is supposed to represent, the distribution of the errors cluster around zero, with a central ‘bump’, since many errors should be small (Figure 2). If representations are fully independent, we should not find any systematic relationship between the unmatched pairs (e.g., response #2 compared to memory item #1; response #3 compared to memory item #1; response #1 compared to memory item #2; etc); for these pairings, the distribution of errors should be uniform—as there was no true relationship between the memory item themselves in this data, and so if responses reflect only the relevant memory item, there should be no relationship between the response and other memory items.

If the responses are not totally independent, but still purely item-based, we should find a mixture of correct reports and ‘swaps’ based on location memory errors (as argued by e.g., Bays et al., 2009; Oberauer & Lin, 2017). This would lead to a small “bump” near 0 for all of the non-target items (since their location is random with respect to the target item, all errors should be random except for ‘swaps’ that generate a response near the non-target; see Bays et al., 2009). By contrast, if clusters, chunks and gist are used to represent the displays (e.g., Brady & Alvarez, 2011; Nassar et al., 2018; Son et al., 2020), we should find systematic relationships between the items, and these relationships could be considerably more complex than simply swaps, leading to error distributions that are not just equal central bumps for all responses.

For each memory response, paired with each memorandum, we quantified whether there were any systematic relationships between memoranda and responses by computing the percentage of observations within 90 degrees of the memory color. We used this metric because since our “null hypothesis” is a uniform distribution, it is clearly interpretable and also sensitive to both more and fewer responses near the relevant memoranda, unlike other straightforward error metrics. In particular, if there is no relationship, 50% of responses should be in this range (−90 to 90), and 50% outside this range (i.e., falling from −180 to −90 and 90 to 180), as is the case in a true uniform distribution. Systematic relationships are defined by the percentage that reliably has either more or less than 50% of responses in this window.

**Figure 3.**
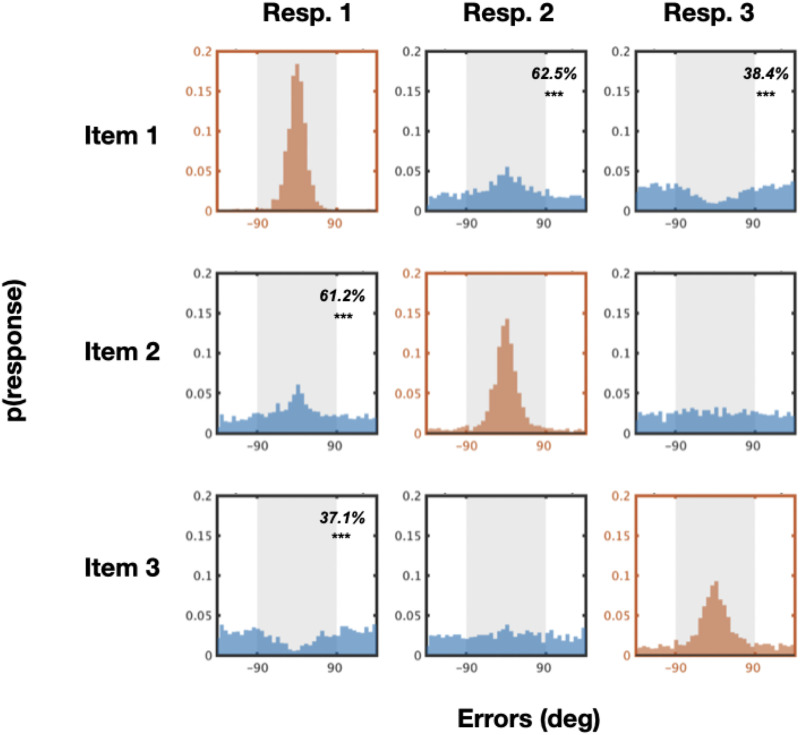
**Set size 3.** Error distributions for all possible responses paired with all possible target memory items. If there are no systematic relationships between memory items, one would expect to see more errors within the ±90° range (>50%) only along the diagonal axis. In contrast to this, we found many systematic relationships between unmatched pairs in both directions (both more or less errors within ±90° range).

**Figure 4.**
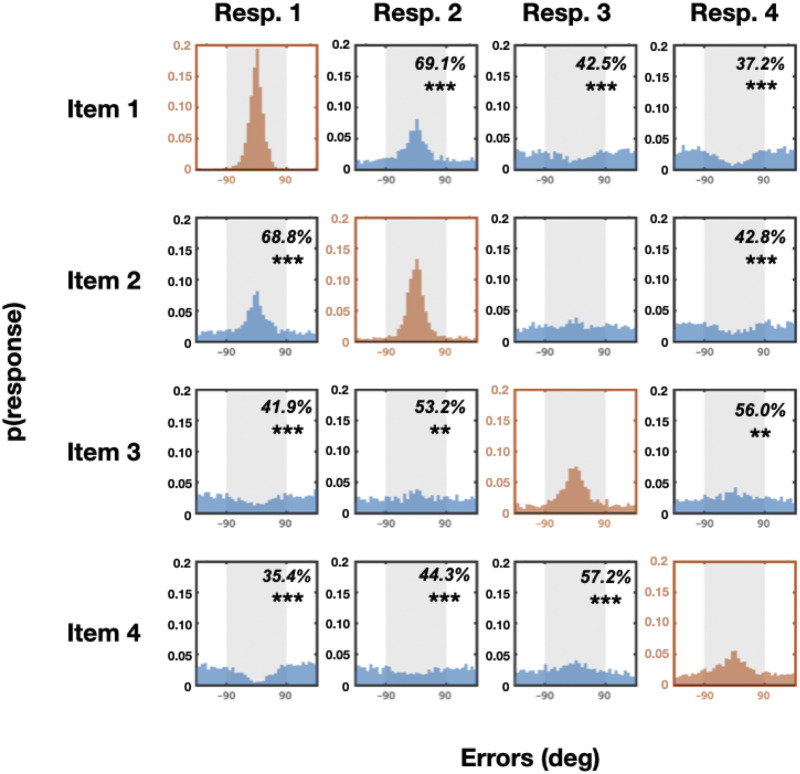
**Set size 4.** Error distributions of all possible responses from all possible target memory items. If there are no systematic relationships between memory items, one would expect to see more errors within the ±90° range (>50%) only along the diagonal axis. By contrast, we found many systematic relationships between unmatched pairs in both directions (both more or less errors within ±90° range).

**Figure 5.**
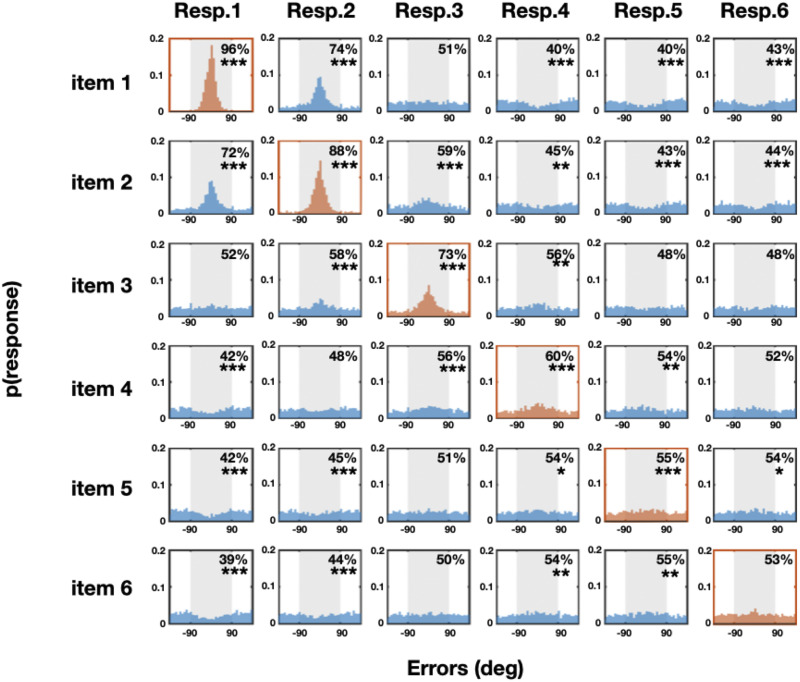
**Set size 6.** Error distributions of all possible responses from all possible target memory items. If there are no systematic relationships between memory items, one would expect to see more errors within the ±90° range (>50%) only along the diagonal axis. In contrast, we found many systematic relationships between unmatched pairs in both directions (both more or less errors within ±90° range).

**Figure 6.**
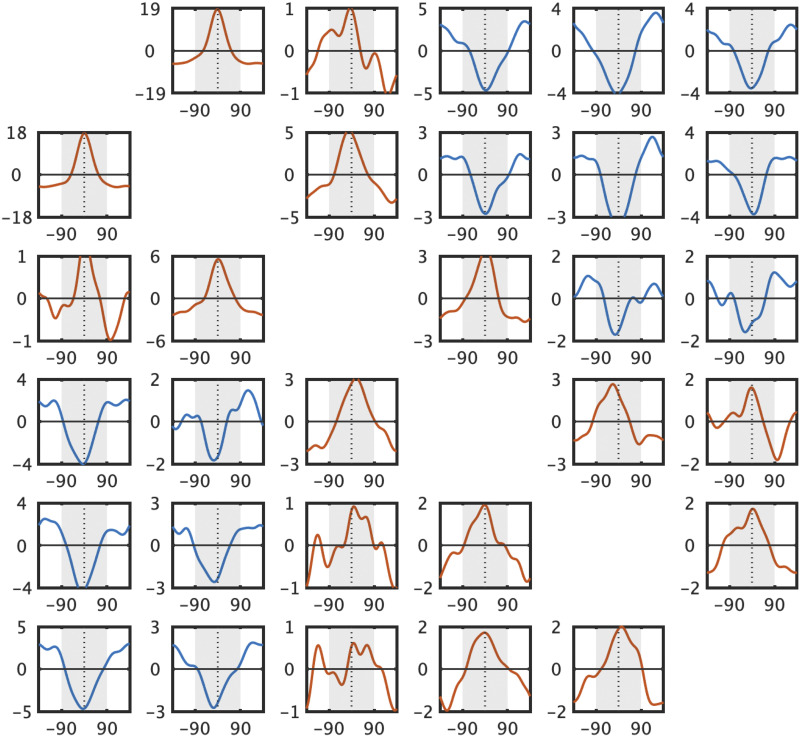
Set size 6, subtracted from uniform to make the trends more visible for off-diagonal locations. Either an overrepresentation of central responses (red) vs. an underrepresentation (blue) are clearly visible in all the off-diagonal positions.

The results are shown in Figures 3, 4, 5, and 6. Overall, we find strong interactions among nearly all of the items at all of the set sizes. These interactions are far richer than a simple ‘swap’ prediction or a simple guessing strategy, in many cases being the exact opposite of what would be predicted from ‘swap’ models (e.g., Bays et al., 2009; Oberauer & Lin, 2017). In particular, we find systematic attraction and repulsion to other items (e.g., not the item being probed), and find these occur even for strongly represented items. Thus, these results suggest rich interactions between the items that go above and beyond simple guessing or swapping.

At set size 3, where conceivably according to ‘slot-like’ models participants should have no problem representing each item as an independent unit and where ‘guessing’, if it exists, is minimal, we nevertheless find strong non-independence: Responses to the first and second item are positively related, whereas response 3 is anti-correlated to memory item #1. That is, the second response participants give is systematically *similar* to the first item they reported, whereas the third response people give is systematically *dissimilar* from the first memory item. Note that while systematic similarity could be caused, in theory, by location confusions (e.g. ‘swaps’), systematic dissimilarity is not consistent with any location-based confusion story. Models that take either swaps to all other items or swaps based on location to be the primary driver of interactions between items are strongly incompatible with this data (e.g., Bays et al., 2009; Oberauer & Lin, 2017). A unified theory of both is necessary to account for this data given the presence of both systematic similarities and dissimilarities.

At set size 3 there are, naturally, systematic relationships between pairs where the items are plotted against their intended memory item (*N* vs. *N*; Figure 3 in red colors; % of responses within 90 deg. are 97.8%, 90.2%, and 76.6% with *t* = 58.1, 28.4, 12.6, Cohen’s *d* = 12.4, 6.1, 2.7, *p* < 0.001 after Bonferroni correction for response 1 paired with item 1, response 2 paired with item 2, response 3 paired with item 3 respectively). More importantly, however, there are also systematic relationships in the majority of pairs where the responses are plotted against other memory items as well. In general, the “near” unmatched response-vs.-memory pairs (*N* vs. *N* − 1 or *N* + 1, e.g., response 2 paired with item 1, response 1 paired with item 2, response 2 paired with item 3 and so on) have significantly more responses closer to the memory colors (mean of 57.2%, *t*(21) = 8.7, Cohen’s *d* = 14.7, *p* < 0.001 after Bonferroni correction). On contrary, the “far” unmatched response-vs.-memory pairs (*N* vs. *N* − 2 or *N* vs. *N* + 2; e.g., the rest of the pairs that are not classified as “near” unmatched) have significantly *fewer* responses closer to the memory colors (mean of 37.7%, *t*(21) = 10.4, Cohen’s *d* = 6.8, *p* < 0.001 after Bonferroni correction). Looking at individual pairings alone, there are significantly more % of responses closer to unmatched memory colors for response 2 paired with item 1 and response 1 paired with item 2 (Figure 3 in blue; 62.5% and 61.2%, *t* = 8.0, 6.8, Cohen’s *d* = 1.7, 1.4, *p* < 0.001 after Bonferroni correction respectively). There are also significantly fewer responses close to unmatched memory colors for response 3 paired with item 1, and response 1 paired with item 3 (38.4%, 37.1%; *t* = 6.2, 12.9, Cohen’s *d* = 1.3, 2.8, *p* < 0.001 after Bonferroni correction).

At set size 4, there are, again, as expected by all theories, systematic relationships between matching memory-response pairs (*N* vs. *N*; Figure 4 in red colors; % of responses within 90° are 97.2%, 88.3%, 74.7%, and 63.8% with *t* = 79.9, 26.8, 12.9, and 7.3; Cohen’s *d* = 17.0, 5.7, 2.7, 1.6; *p* < 0.001 for response 1 paired with item 1, response 2 paired with item 2, response 3 paired with item 3, response 4 paired with item 4 respectively). However, in contrast to theories of independent representations and of swap-only theories, there are also significant relationships between almost all unmatched pairs except response 3 paired with item 2 and response 2 paired with item 3. Similar to the findings at set size 3, there are significantly more responses closer to the memory colors in near unmatched pairs (59.3%, *t*(21) = 19.5, Cohen’s *d* = 26.5, *p* < 0.001 after Bonferroni correction) and fewer responses closer to the memory colors in far unmatched pairs (40.6%, *t*(21) = 13.4, Cohen’s *d* = 12.4, *p* < 0.001 after Bonferroni correction). Specifically, there are larger percentages of responses closer to unmatched memory colors for response 1 paired with item 2, response 2 paired with item 1, response 2 paired with item 3, response 3 paired with item 2, response 3 paired with item 4, response 4 paired with item 3; 68.8%, 69.1%, 53.2%, 51%, 57.1%, 55.8%; *t* = 10.7, 10.3, 2.9, 1.5, 5.7, 3.6; Cohen’s *d* = 2.3, 2.2, 0.6, 0.3, 1.2, 0.8; *p* < 0.001, *p* < 0.001, *p* = 0.05, *p* > 0.05, *p* < 0.001, *p* < 0.01 after Bonferroni correction respectively). There is a significantly lower percentage of responses closer to the unmatched memory colors for response 1 paired with item 3, response 1 paired with item 4, response 2 paired with item 4, response 3 paired with item 1, response 4 paired with item 1, and response 4 paired with item 2 (41.8%, 35.4%, 44.2%, 42.5%, 37.0%, 42.8%; *t* = 7.7, 10.3, 4.9, 6.9, 8.9, 4.3; Cohen’s *d* = 1.6, 2.2, 1.0, 1.5, 1.9, 0.9; *p* < 0.001, *p* < 0.001, *p* < 0.001, *p* < 0.001, *p* < 0.001, *p* < 0.01 after Bonferroni correction respectively).

At set size 6, there are systematic relationships between most of the matched memory-response pairs (Figure 5 in red colors and Table 1 along the diagonal). There are also significant relationships between most of the unmatched pairs (see Figure 6 and Table 1 off the diagonal). Specifically, there are larger percentages of responses closer to unmatched memory colors for response 1 paired with item 2, response 2 paired with item 1, response 2 paired with item 3, response 3 paired with item 2, response 3 paired with item 4, response 4 paired with item 3. There are significantly less % of responses closer to unmatched memory colors for response 1 paired with item 4, response 1 paired with item 5 response 1 paired with item 6, response 2 paired with item 5, response 2 paired with item 6, response 4 paired with item 1, response 5 paired with item 1, response 5 paired with item 2, response 6 paired with item 1, response 6 paired with item 2 (see Figure 6 and Table 1 off the diagonal).

**Table 1.**
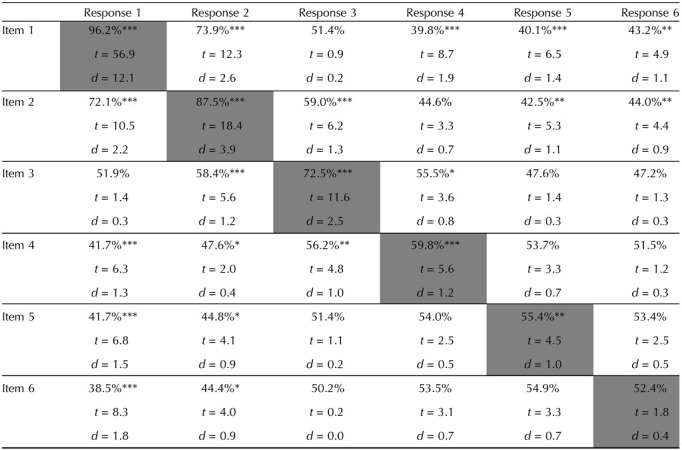
Relationships at set size 6 between items and responses, focusing on the percentage of responses close vs. far from the target item (low numbers mean fewer responses near that item than would be expected; high numbers mean more).

* Bonferroni corrections were performed for all matched and unmatched pairs separately.

Thus, we find a complex set of relations between items is present in this data, far from what would be expected if items are represented and reported independently or if swap errors, or other item-based guessing strategies, were solely responsible for item-response relationships.

#### Biases Toward and Away From Other Items.

Another way to test for relationships between each response and each memory item is to see whether responses to the matched memory item systematically shift away or toward unmatched memory items. Previously, we have shown that people systematically distorted memory for individual items, e.g., at set size 2, similar items repel each other to increase distinctiveness (Chunharas et al., 2022). Does the same occur for entire “clusters” of items?

To visualize this effect, we can plot the error distribution of the matching pairs of responses and memoranda (e.g., response 1 item 1) as if the unmatched memory item of interest (e.g., item 2) is always on the counter-clockwise side of the color wheel. If the unmatched item does not have any influence, the error distribution should remain symmetric. On the other hand, the error distribution should skew counterclockwise or clockwise if the responses are attracted or repelled from the unmatched memory item of interest. To quantify this effect, we calculated the differences between the percentages of clockwise vs. counterclockwise error. The differences in percentages of responses more or less than 0% indicate systematic repulsion and attractions biases respectively (e.g., positive means more repulsion: see also Chunharas et al., 2019, 2022).

**Figure 7.**
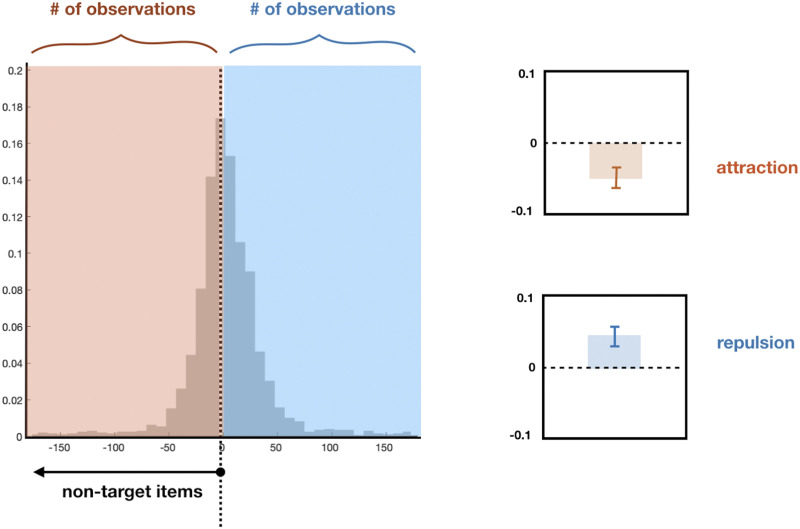
Quantification of systematic attraction and repulsion biases. We can plot the error distribution as if the non-target colors of interest were always on the counter-clockwise of the color wheel. More responses on the counterclockwise side (red) thus indicate attraction and more on the clockwise side (blue) thus indicate repulsion. We can then take a difference score, such that less than 0% vs. more than 0% indicate attraction and repulsion biases respectively.

**Figure 8.**
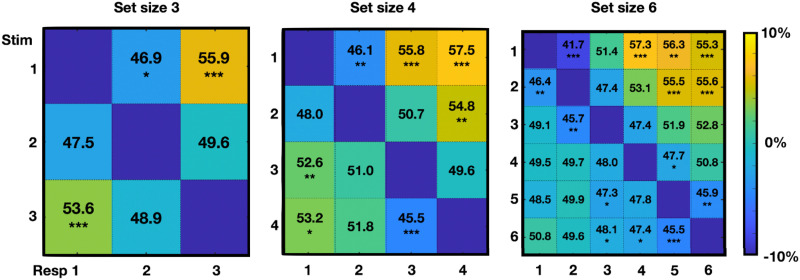
Heat map of repulsion (hot colors) and attraction (cold colors) in the Adam et al. (2017) data. The first few responses and, separately, the last few responses attract toward each other; while the first few responses repel from the last few responses. *% of responses toward the target item.

Overall, we find that the first few responses and, separately, the last few responses attract toward each other; while the first few responses repel from the last few responses (see Figure 7 for our definition of attraction and repulsion). At set size 3 (Figure 8), we found significant attraction biases in near unmatched pairs (48.3%, *t*(21) = 2.7, Cohen’s *d* = 16.3, *p* < 0.05 after Bonferroni correction) while found significant repulsion biases in far unmatched pairs (54.6%, *t*(21) = 5.6, Cohen’s *d* = 13.9, *p* < 0.001 after Bonferroni correction). For individual item-response analyses, we found biases for response 3 and item 1, response 1 and item 3; 55.7%, 53.6%; *t* = 4.0, 3.0; Cohen’s *d* = 0.9, 0.6; *p* < 0.01, *p* < 0.05 after Bonferroni correction.

At set size 4, we again found significant attraction biases in near unmatched pairs (48.4%, *t*(21) = 3.0, Cohen’s *d* = 19.7, *p* < 0.05 after Bonferroni correction) while found significant repulsion biases in far unmatched pairs (54.2%, *t*(21) = 6.7, Cohen’s *d* = 18.3, *p* < 0.001 after Bonferroni correction). For individual pairs of items and responses, we found systematic biases for response 2 and item 1, response 3 and item 1, response 4 and item 1, response 4 and item 2, response 3 and item 4; 46.0%, 55.7%, 57.5%, 54.9%, 45.5%; *t* = 3.6, 4.7, 5.6, 3.7, 3.9; Cohen’s *d* = 1.0, 1.2, 0.8, 0.9; *p* < 0.05, *p* < 0.01, *p* < 0.001, *p* < 0.05, *p* < 0.01.

At set size 6, we also found significant attraction biases in near unmatched pairs (46.4%, *t*(21) = 6.6, Cohen’s *d* = 17.9, *p* < 0.001 after Bonferroni correction) while found significant repulsion biases in far unmatched pairs (51.5%, *t*(21) = 3.4, Cohen’s *d* = 25.0, *p* < 0.01 after Bonferroni correction). For individual item analyses, we found systematic biases for response 2 and item 1, response 4 and item 1, response 5 and item 1, response 6 and item 1, response 5 and item 2, response 6 and item 2, response 5 and item 6; see Table 2 for statistics.

**Table 2.**
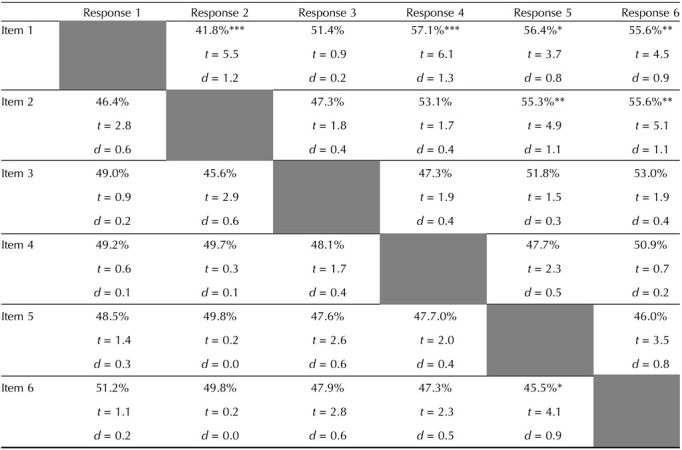
Bias toward vs. away from the item in the response. Numbers greater than 50% mean responses are repelled from that item; numbers below 50% show attraction.

* Bonferroni corrections were performed for all matched and unmatched pairs separately.

#### TCC-Motivated Clusters and Chunks.

The above analyses show that both in terms of the overall tendency to report similar vs. dissimilar colors, and in terms of the shift of response distributions, participants do not store and report the items’ independently of each other in this dataset. What representational structure are people using to store and report these displays?

It has been shown that when subjects need to remember large amounts of information, they extract some form of ‘gist’ or ‘ensemble’ information and use it to help them maintain individual information in a hierarchical fashion (e.g., Brady et al., 2011). Previous studies have shown that the error responses of each memory item were attracted toward such gist-based summary information (Brady & Alvarez, 2011; Orhan & Jacobs, 2013). However, in displays where the structure does not encompass all items—like in the current, randomly generated displays—it is unclear whether such attraction applies to all memory items or only to the memory items that are similar to the gist. On the one hand, it is possible that all responses might be attracted toward the overall global gist color. On the other hand, some responses might systematically shift toward the gist color while others might have no systematic bias or even repel from the gist color (for example, if ‘outliers’ are segregated before summaries are computed; Epstein et al., 2020; Haberman & Whitney, 2010).

The idea that not all items would be attracted to a single “gist” is consistent with the possibility that on displays that are randomly-generated and so do not all have a single “gist”, participants may “chunk” the display into similarity-based clusters (e.g., Nassar et al., 2018; Son et al., 2020).

To test these ideas in the current dataset, and ground them in a computational model of chunking, we propose a new chunking model based on the psychophysical similarity function of Schurgin et al. (2020). This model is closely related to the TCC model of working memory (Schurgin et al., 2020) and the application of this TCC model to explain ensemble perception (Robinson & Brady, 2023). For simplicity, the instantiation of the model we use here considers the color information independent of location information for chunkability, although surely location must play some role (e.g., if all red items are on the left and all blue on the right, this will surely reduce location uncertainty compared to if the items are intermixed).

**Figure 9.**
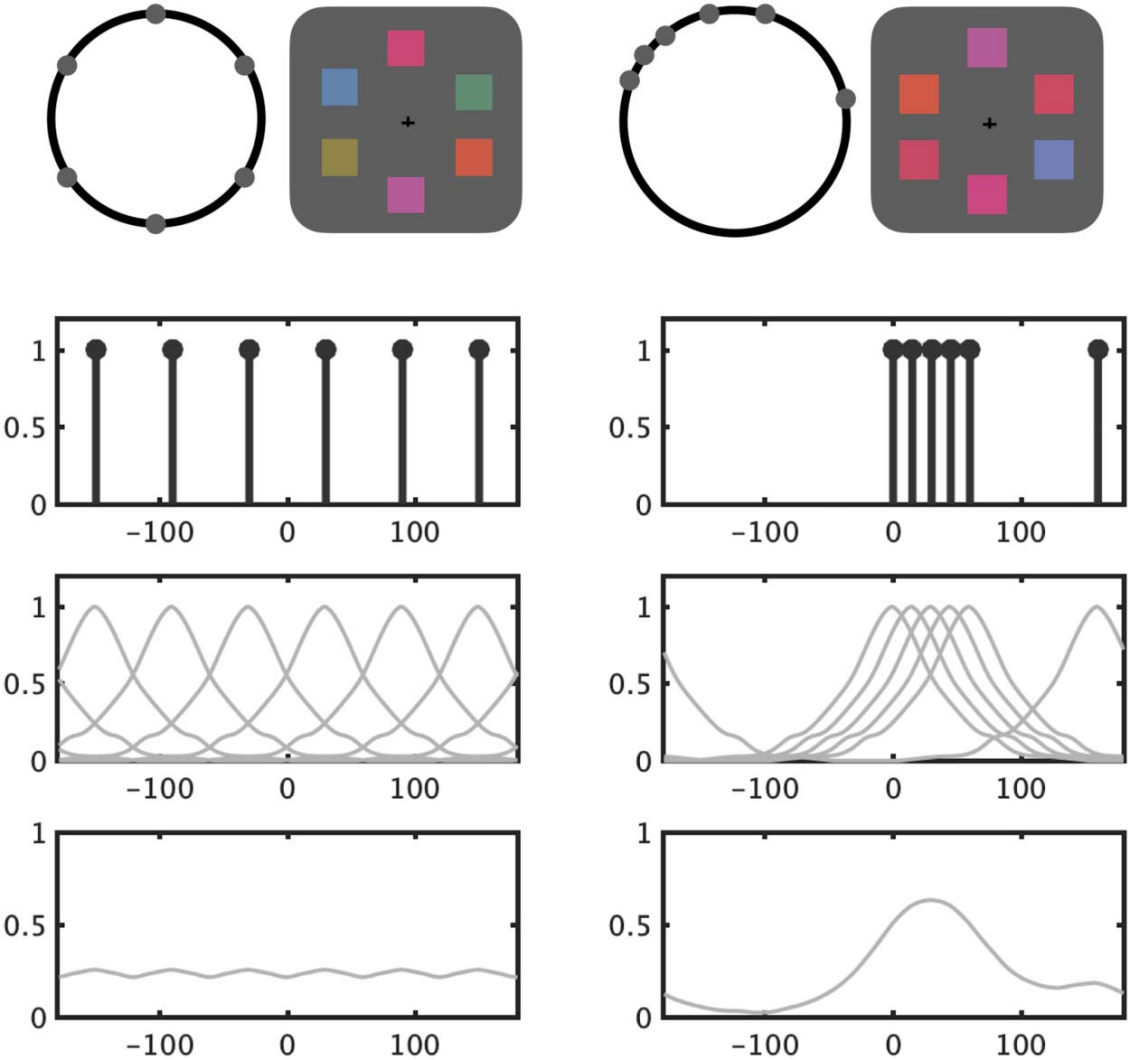
**Examples of chunkability and quantifying this chunkability**. Examples of two hypothetical displays that scored as the least chunkable (left) and the most chunkable (right) in the Adam et al. (2017) data. The top row shows how the six memory colors were distributed on the color wheel. The second row represents the distribution of memory items with the color space unfolded along the *x*-axis rather than represented as a circle. The third row represents the psychological similarity kernel that is centered on each of these colors. The last row represents the sum of all kernels from the previous row, showing the general pattern of activation expected from the entire display (in line with the ensemble model of Robinson & Brady, 2023). Here, we take the height of the highest peak to indicate how chunkable the display is, with the peak location indicating where the gist color is for a given chunk.

The model is straightforward: in line with the TCC model of memory (Schurgin et al., 2020), each color is assumed to generate activation not only in representations of the exact color that was shown but also in similar colors (e.g., orange also causes some activation of dark yellow), according to a fixed psychophysical similarity function (Schurgin et al., 2020). These activation profiles for each color are then summed together to give an overall sense of what colors are present in the display (see also Robinson & Brady, 2023, for a similar model applied to ensemble report tasks). This is then normalized to give a sense of the “chunks” in the display. If all the items are quite distinct, there will be minimal overlap in their psychophysical similarity function and this summed representation will be quite flat once normalized (e.g., Figure 9, left). If many items are close in color space, this representation will be quite peaky (e.g., Figure 9, right). The number of peaks present in this summed representation naturally tracks “how many” chunks are formed (6 on the left, 2 on the right), and the height of these peaks tracks how strongly these chunks are present in the display (e.g., the display on the right has 1 strong chunk and 1 weak chunk). The peak color in this representation is the single best “gist” color for the display, once psychophysical similarity is taken into account.

While grounded in the TCC framework, this model is similar to what would be expected from considering neural tuning functions: seeing red causes activation in neurons that code for not only red but nearby colors (e.g., Bays, 2015), leading to a set of activations broadly in line with the psychophysical similarity function; and summing these is broadly consistent with considering what tuning functions would be activated in the entire population of neurons responding to the display, independent of location.

We find this chunking model captures important information about performance in the Adam et al. (2017) task. We focus on set size 6 from Adam et al. (2017) in these analyses, since it provides the richest set of chunking data, but then consider other set sizes with the same chunking model below.

#### Effect of Chunkability on Performance for Items.

**Figure 10.**
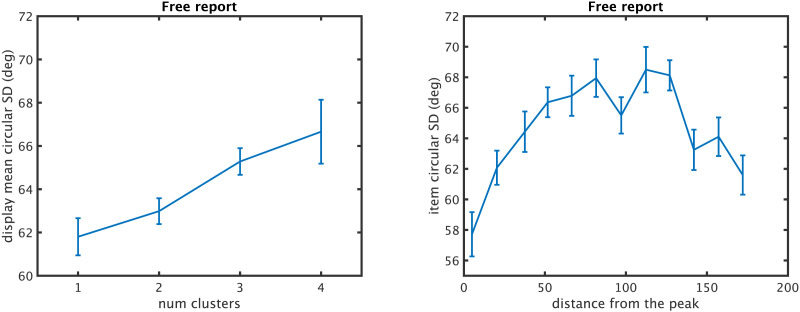
Relationship between the chunkability of the display and performance for the entire display (left) and particular items (right), in free response data of Adam et al. (top) and random response data (bottom).

We first used this model to assess overall performance on individual displays, and then to assess performance on items as a function of how close to the ‘gist’ color they were. We found that displays with fewer clusters are better remembered, with lower error on average in reporting all of the items from such displays (Figure 10, left). This is true in both free report, which we have focused on so far, where participants are free to respond in any order (Figure 10, top; *F*(3, 63) = 3.95, eta^2^ = 15.8, *p* < 0.05) and in random report, which is more like a typical working memory paradigm where participants must respond to particular items in the order the experimenter cues (Figure 10, bottom; *F*(3, 63) = 4.65, eta^2^ = 18.1, *p* < 0.01).

We also found an effect of the gist color on memory for particular items. Items closest to the “gist” (the highest peak in the summed psychophysical function) were more accurately remembered than those further from this peak, consistent with participants using the gist to structure their memory for the display. This was true in both free response data (*F*(11, 231) = 5.93, eta^2^ = 22.0, *p* < 0.001) and random report (*F*(11, 231) = 2.85, eta^2^ = 11.9, *p* < 0.01).

#### Systematic Biases and Chunks/Clusters.

Previous studies have shown that when items are represented as part of the same “unit”, they tend to attract toward each other and be reported as more similar than they really were (e.g., Brady & Alvarez, 2011); whereas when items are represented as part of separate “units”, they tend to repel each other and be reported as less similar than they really were. For example, Chunharas et al. (2022) found that when items are represented as part of a single unit—for example, 4 very similar colors—they tend to attract each other, whereas when item information is strong enough to be segregated, as when 2 items are present and both can be individually represented well, the items tend to repel each other. Similarly, Lively et al. (2021) showed that when memory is strong and items can be strongly represented as individuals, items tend to repel each other; whereas when memory is weak, items tend to attract toward each other (Lively et al., 2021).

**Figure 11.**
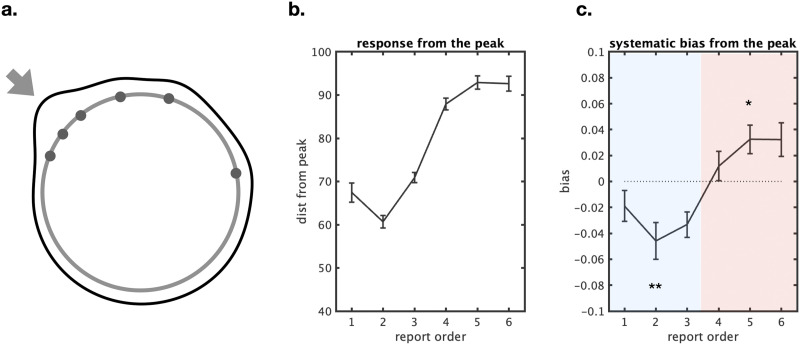
Relationship between the order of responses and the gist color. (a) An example of the relationship between memory items (circles) and the gist color (arrow) on a circular color space. (b) We found that subjects responded closer to the gist color in the earlier responses. (c) Moreover, the earlier responses (in blue shaded region) were systematically biased toward the gist colors while the later responses (in red shaded region) repelled from the gist colors.

To test this hypothesis in the context of this chunking model—as opposed to considering the entire display part of a single chunk, as done in previous work (e.g., van den Berg et al., 2014)—we computed the systematic biases and color distances from each response to the global gist color, focusing on more chunkable trials (less chunkable trials did not have clear gist colors). We define bias as the difference between the correct answer and the reported answer, and we flip each trial so that negative always means “towards the gist” and positive always means “away from the gist”. We found different systematic biases for different responses in terms of report order (*F*(5, 105) = 63.8, eta^2^ = 75.2, *p* < 0.001; Figure 11). Attraction biases toward the global gist were found in earlier reports (*t*(21) = 3.7; Cohen’s *d* = 0.8, *p* < 0.01 for the average of report 1–3, corrected for multiple comparison) and repulsion biases were found in later reports (*t*(21) = 2.9; Cohen’s *d* = 0.6, *p* < 0.05 for the average of report 4–6, corrected for multiple comparison).

#### The Direction of Systematic Error Depends on the Chunk-Ability of the Displays.

**Figure 12.**
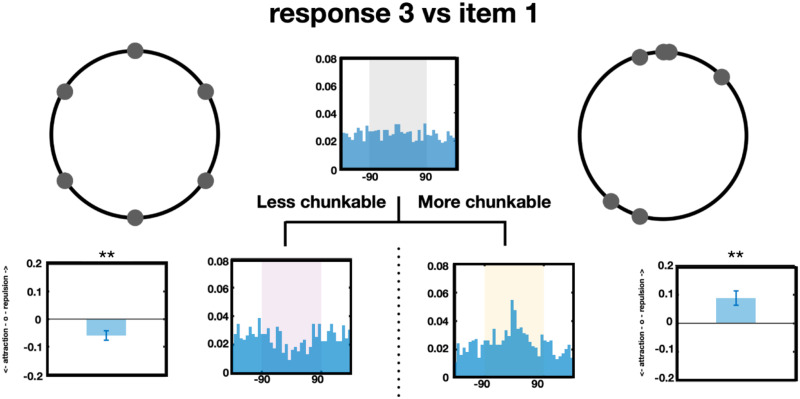
The systematic biases depend on how chunkable the displays are. The third responses were repelled from the first memory items in less chunkable trials and attracted toward the first responses in more chunkable trials.

On the surface, in our initial analyses (e.g., Figures 5 and 6) of the set size 6 data, the third responses did not appear to have a systematic relationships with the first memory item. To address why, in terms of this unifying framework of chunking, we entertained the possibility that some of the third responses went toward while some went away from the first memory item, and that which occurred depends on whether the displays were more or less *chunkable* respectively. To test this hypothesis, we quantified how chunkable each display was by our TCC-based chunking model. Then, we did a median split (e.g., split the data into the top and bottom half, with equal trial numbers in each) and did the same analysis for more and less chunkable displays separately. We found that the third responses tended to go toward the first memory item in more chunkable trials (*t*(21) = 3.45, Cohen’s *d* = 5.4, *p* < 0.01 after Bonferroni correction) and tended to go away from the first memory item in less chunkable trials (*t*(21) = 3.46, Cohen’s *d* = 4.9, *p* < 0.01 after Bonferroni correction; Figure 12). This shows how considering the interactions between items in displays without carefully taking into account the entire chunk structure of the display may be misleading.

### Summary

Overall, and in contrast to claims of independent encoding of a fixed number of items in this dataset, we find evidence for clustering (e.g., treating multiple items as a group, with both attraction to the group gist and repulsion from other group’s gists), chunking (of which clustering is a particular form based on perception), and non-item-based representations (including clustering). In particular, people tend to be more accurate in reporting colors that are close to the “gist” color of the display, and these first responses are systematically attracted toward each other (e.g., reported closer together in color space than the items truly had been). The number of items that are grouped depends on the chunkability of the display (more chunkable display associates with more grouping of responses). Examining the last items people report on a given display also reveals they are not random responses. Instead, the later responses (including the last) are systematically repelled from the earlier ones. Thus, rather than encoding items independently, memoranda are compressed by combining and separating items in relation to each other, using clusters. A simple model of chunking based on the TCC similarity function can be used to reveal these clusters and to understand the pattern in this data, consistent with how the TCC model has been applied to understanding ensemble perception (Robinson & Brady, 2023). Next, we turn to other datasets, fitting the same chunking model and examining how widespread these phenomena are.

## EXPERIMENTAL RE-ANALYSIS 2: COLOR AND ORIENTATION REPORT FROM OPEN DATASETS

The Adam et al. (2017) dataset is unique in requiring participants to respond to each item on each trial, and allowing free report where in some conditions, participants choose the order items to report. This could in theory have exacerbated people’s tendency to rely on chunking and clustering. While our analyses of these results and showing of systematic attraction and repulsion in all responses in this dataset is still critical, since the Adam et al. (2017) work has been central to arguments for fixed item limits, these properties of this dataset may limit some aspects of the generality of the analyses presented in part 1. Thus, in the next reanalysis, we considered data from a wide variety of other open datasets of more standard visual working memory situations.

### Methods

We next performed a re-analysis of the data made available by van den Berg (2014). This was 10 datasets. They were described as shown in Table 3.

**Table 3.** A table showed stimulus features, set sizes, stimulus duration (in millisecond), memory delay interval (in millisecond) and number of subjects from 10 experiments that were renanalyzed. The information was adapted from van den Berg (2014). We included the work of Anderson and Awh because retractions of this work focused on the individual difference analyses, which are not relevant to our modeling, and because this work appeared consistent with all the other studies.

	**Article**	**Feature**	**Set sizes**	**Stimulus (ms)**	**Delay (ms)**	**Subjects**
1	Wilken and Ma (2004)	Color (wheel)	1, 2, 4, 8	100	1,500	15
2	Zhang and Luck (2008)	Color (wheel)	1, 2, 3, 6	100	2,000	8
3	Bays et al. (2009)	Color (wheel)	1, 2, 4, 6	100	1,000	12
4	Anderson et al. (2011)	Orientation (360 deg)	1, 2, 3, 4, 6, 8	200	1,000	45
5	Anderson and Awh (2012)	Orientation (180 deg)	1, 2, 3, 4, 6, 8	200	1,000	23
6	Anderson and Awh (2012)	Orientation (360 deg)	1, 2, 3, 4, 6, 8	200	1,000	23
7	van den Berg et al. (2012)	Color (scrolling)	1, 2, 3, 4, 5, 6, 7, 8	110	1,000	13
8	van den Berg et al. (2012)	Color (wheel)	1, 2, 3, 4, 5, 6, 7, 8	110	1,000	13
9	van den Berg et al. (2012)	Orientation (180 deg)	2, 4, 6, 8	110	1,000	6
10	Rademaker et al. (2012)	Orientation (180 deg)	3, 6	200	3,000	6

### Results

These data do not allow as rich an analysis of the relations between responses and items as that allowed by Adam et al. (2017)’s data (e.g., Figures 3, 4, 5, 6), since only a single item is reported per trial. Nevertheless, we can take the same TCC-based chunking model we developed from that data and apply it to these data to assess its generality. We use different similarity functions for color and orientation, in both cases using existing psychophysical similarity data (see Cohen et al., 2023 for a report of the orientation similarity data and Schurgin et al., 2020 for the color data).

**Figure 13.**
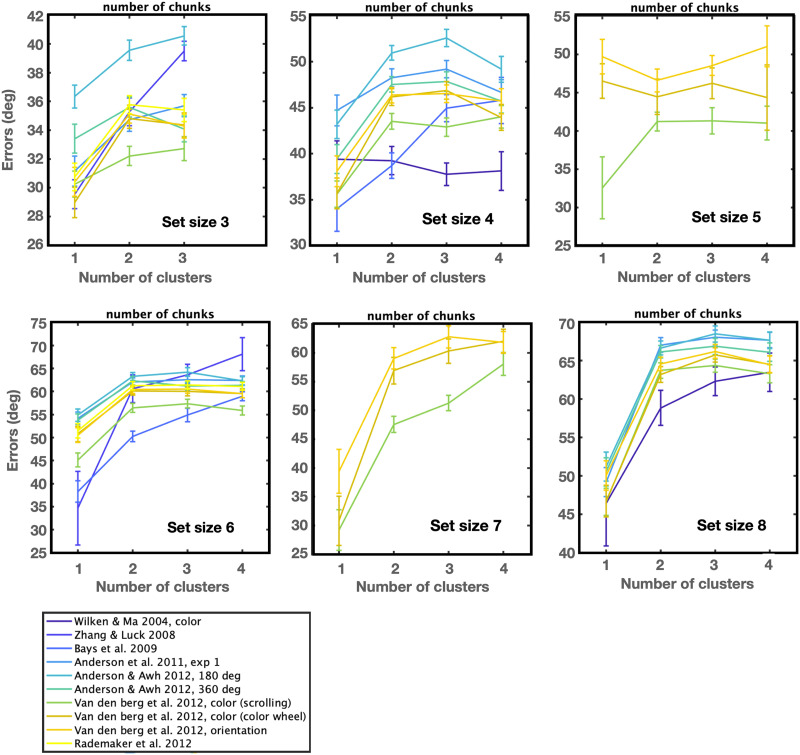
The number of chunks influences performance to a significant extent at nearly every set size in nearly every dataset. Note that we included Anderson’s papers in this analysis and found consistent results with the majority of the studies.

Examining the datasets independently, we find a large effect of the number of chunks on performance in almost every dataset at almost every set size, as shown in Figure 13. In particular, displays with fewer chunks are on average remembered better at almost all set sizes in almost every experiment (Set size 3–8 with *F*(2, 272) = 11.1; eta^2^ = 0.08, *p* < 0.001, *F*(3, 446) = 15.9; eta^2^ = 0.1, *p* < 0.001, *F*(3, 93) = 2.6 eta^2^ = 0.02; *p* > 0.05, *F*(3, 441) = 50.6; eta^2^ = 0.3, *p* < 0.001, *F*(3, 91) = 40.74; eta^2^ = 0.6, *p* < 0.001, and, *F*(3, 410) = 83.3; eta^2^ = 0.4, *p* < 0.001 respectively).

**Figure 14.**
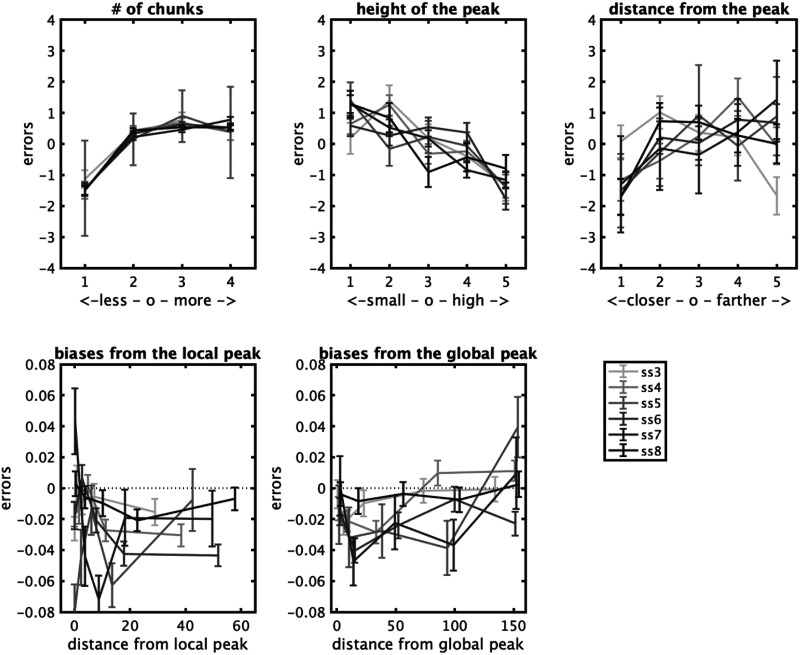
Summary statistics across datasets, plotted in *z*-scores of the error to make them comparable across set sizes (ss). Error decreases when there are fewer chunks, depends on the chunkability of the display (height of the peak), and the distance of a given item from the peak. Items are attracted to local peaks and to some degree from global peaks.

**Figure 15.**
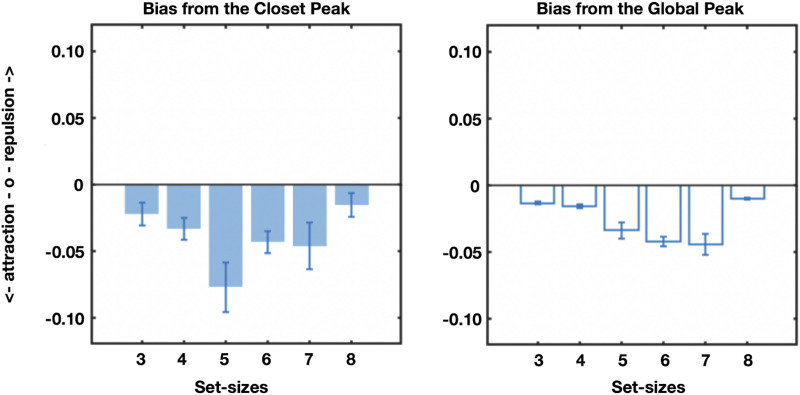
Biases toward local and global peaks.

Collapsing across datasets to examine the effects of chunking and set size independent of datasets, we find that items have less error when there are fewer chunks in the chunking model (Figure 14; statistics shown in Figures); when the peak is more pronounced (e.g., the display is more chunkable), and when the items are close to the peak or the ‘gist’ (Figure 14), all consistent with the predictions of the model we developed in the Adam et al. (2017) data. There are also systematic biases present here, as in the data reported by Adam et al. (2017): Items are attracted toward the nearest local peak in the chunking model systematically, and in a way that depends on their distance from that peak. Items are also on average attracted to the global peak, although this is hard to disentangle, in this analysis, from them being likely to be part of that peak’s chunk on average (Figure 15). These results are similar to the proposal of Chunharas et al. (2022) about attraction and repulsion depending on the distance of the item from the gist.

What about repulsion from items in other clusters, as observed between the first few and last few items reported in the Adam et al. data? It is hard to quantify this for arbitrary displays of arbitrary set sizes with arbitrary number of chunks, since there is no fixed relationship between chunks that can be used.

**Figure 16.**
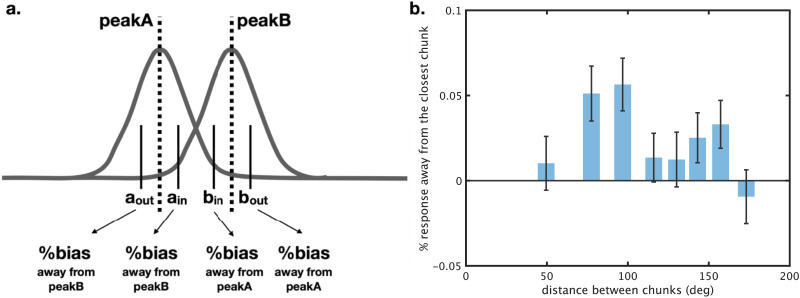
Items are repelled if they are in distinct chunks, in two-item chunk displays. When the distance between two chunks were discretized into 8 bins, the repulsion biases were particularly strong when the items were a moderate distance away from the other chunk. This non-monotonic relationship between repulsion magnitude and feature similarity mirrors the pattern observed in the two-item display from Chunharas et al. (2022).

Thus, to examine this, we focus on displays that our chunking model says have 2 clear chunks in them, and no more or less. 37% of trials, or 48,928 trials total, had 2 clear chunks, allowing a relatively generalizable test of this hypothesis. On these displays, we can select items on both sides of the ‘peak’ in each chunk, and ask if these items are repelled from each other, as suggested by the generalization of the Chunharas et al. (2022) model of items to entire chunks. As shown in Figure 16, we find that, in general, the responses to these items are repelled away from the other chunk (*t*(163) = 4.2, Cohen’s *d* = 0.3, *p* < 0.001). The magnitude of repulsion also peaks when chunks are not too close nor too far away from each other, as would be expected a priori based on the case of two memory items, where this pattern is generally observed (Bae & Luck, 2017; Chunharas et al., 2022; Golomb, 2015) (*F*(7, 1141) = 2.18, eta^2^ = 1.32, *p* < 0.05). Thus, like individual items, chunks themselves repel each other and do so most when they are moderately similar in feature space. As a control, we re-ran this analysis but randomly flipped the signs of the errors (e.g., clockwise to counterclockwise and vice versa). This control analysis revealed no such biases, so this bias is not an artifact of subsampling just some of the displays but is based on the systematic patterns in the direction of the errors within a display.

Overall, we find evidence in these datasets for all of the findings we observed in the Adam et al. (2017) data: large effects of chunking on overall performance and performance for individual items that are within well-defined chunks; attraction toward local chunks and repulsion of chunks from each other. These patterns are inconsistent with models based solely on individual items, particularly the tendency to organize displays into chunks that cause greater local attraction when they are stronger, and which repel each other, none of which are compatible with simple swap-based models (e.g., Bays et al., 2009; Oberauer & Lin, 2017).

**Figure 17.**
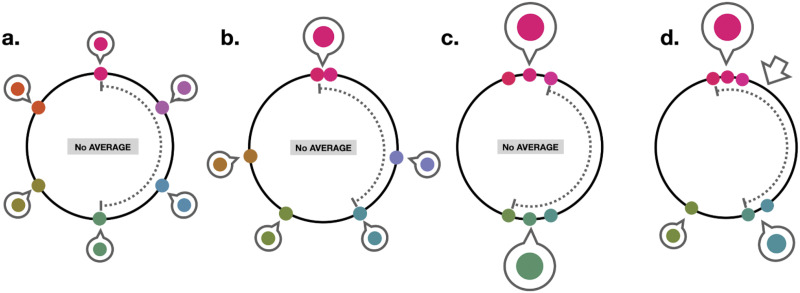
Some displays that would yield different answers if the gist is considered as the “circular average” (open arrow; if available), “range” (dotted line), and “TCC motivated chunking model” (bubbles). In particular, the left 3 displays have no average color (0 resultant vector length), and have large ranges, but differ markedly in their chunkability according to the TCC model, with the left-most having 6 distinct chunks but the third having 2 clear chunks. The right-most display has a mean color that is near the pink cluster, despite it having two clear chunks: the green-ish items would be, according to a circular mean, far from any gist color, even though according to the TCC model they are quite chunkable.

Why has previous work not always found such effects, including a study that used the exact same datasets (van den Berg et al., 2014)? Previous work, with a few exceptions (e.g., Utochkin & Brady, 2020) has used measures that do not capture the structure of the displays in the way our chunkabillity model based on the TCC similarity function does. For example, van den Berg et al. (2014) examined whether there were reliable attractions toward the ‘gist’ in all of these datasets (they referred to this, based on previous work, like Brady & Alvarez, 2011, as the ensemble). They found no such attraction and argue this meant these datasets show evidence of independent item representation. However, they focused on simply whether items are reported closer to the circular mean of all items in the display across all trials, with no attempt to select displays that have a mean color that is possible to perceive and no attempt to understand if the mean color across all colors actually reflected a meaningful gist or ensemble (Figure 17). Our work shows that when analyzed using the TCC-based approach, all of these datasets show reliable attraction effects, in direct contrast to the claim of van den Berg et al. (2014). Other work examining when you get attraction and when you get reduced error from the gist has looked at the range of items on the display or the length of the circular mean vector (Brady & Alvarez, 2015a; Utochkin & Brady, 2020). In the Appendix, we show how those relate to our chunking model. In particular, we find that while the range provides a reasonable proxy for chunkability at set size 3, it fails completely above that. The length of the mean vector captures some aspects of how many chunks are present, but the circular mean is a very poor proxy for where this peak is located. Thus, we suggest that our TCC-based chunking model captures the way people organize these displays in memory more accurately than these other approaches.

## GENERAL DISCUSSION

We have shown that chunking, and attraction and repulsion biases are ubiquitous in visual working memory. We began by focusing on the work of Adam et al. (2017), who have one of the richest publicly available working memory datasets. This dataset was used to make one of the strongest claims, arguing there are limits on working memory performance based on the number of individuated items (Adam et al., 2017). In this data, Adam et al. found that when people are asked to report all 6 items from a set size 6 display in any order they wish, participants appear to have little or no information about the last few items they report (Adam et al., 2017)—a finding they used to argue for fixed item limits, where only 3–4 items are ever stored in memory, with other items completely unrepresented. Other explanations of this pattern of data have since made clear that the same results can be straightforwardly explained by a resource model, with no upper bound on how many items can represented, but where later items in this free report tend to be the lowest confidence items and are thus the most noisy, but even this take on free response data is still through the lens of individuated item representations. In contrast to claims of independent encoding of items, we found strong evidence for clustering, chunking, and non-item-based representations in this data. In particular, people tend to report the colors that are close to the mean color of the display first, and these first responses are systematically attracted toward each other (e.g., reported closer together in color space than the items truly had been). The number of items that are grouped depends on the chunkability of the display (more chunkable being associated with more grouping). Examining the last items people report on a given display also reveals they are not random responses. Instead, the later responses (including the last) are systematically repelled from the earlier ones. Thus, rather than encoding items independently, memoranda are compressed by combining and separating items in relation to each other, using clusters.

We then expanded the analyses and the model that were motivated by Adam et al. (2017)’s data to a wide variety of other datasets made publicly available by van den Berg et al. (2014). We find the same effects are pervasive: displays that are more chunkable are better remembered at nearly every set size in nearly every experiment; these chunks produce systematic attraction and repulsion effects; and differential effects across different items, depending on how well they are captured by such chunks. Overall, we show that no memorandum is an island and each response reflects how that item fits into the whole picture. It is critical to take into account such effects in all models, especially those that attempt to understand how performance changes across set sizes (see below).

### TCC-Motivated Chunking Model

Notably, our findings in the broader group of datasets contrast strongly with van den Berg et al. (2014), who analyzed these same datasets and concluded there was “no evidence for ensemble coding” in visual working memory (e.g., no biases toward the gist). However, several differences explain this discrepancy. van den Berg et al. (2014) tested for bias toward the circular mean of all items, which often provides a poor approximation of actual gist structure, and many displays have no meaningful circular mean or have means that fall far from perceptually coherent clusters. Additionally, they pooled all displays together without considering chunking structure—systematic biases in chunkable displays would be averaged out with non-chunkable displays, yielding null effects overall. Finally, their approach focused on a single global mean rather than the multiple local clusters we show can attract and repel each other.

By contrast, our TCC-based approach identifies gist locations using psychophysical similarity functions and separately analyzes displays based on their chunking structure, revealing the systematic ensemble effects that their methodology was not designed to detect. Thus, using many of the exact same datasets, rather than finding that such effects can be safely treated as a special case, our work instead emphasizes how such effects are ubiquitous across all visual working memory datasets.

Although there is ample evidence to suggest the presence of systematic biases and interactions between items, the underlying representation of the ensemble, gist or of separate chunks has remained unclear. Here, we proposed a model based on the TCC model of visual working memory (Schurgin et al., 2020). Some previous work has posited that the gist is the “average” of the group (e.g., van den Berg et al., 2014) but this approach failed to uncover the pervasive inter-time systematic biases we observe here. Why? One can easily imagine a condition where the display is highly chunkable but has little or no clear “average”. For example, two 3-item chunks that are 180 degrees apart, or three 2-item chunks 120 degrees apart on the color wheel, would each produce no average but be clearly chunkable (see Appendix for a systematic exploration of this issue). Another proposal has quantified the gist as the “range” of the items (Utochkin & Brady, 2020), in other words, how much of the color or orientation space is covered by the items. Despite its simplicity, one can also imagine two conditions with the exact same range but different chunkabilities. For instance, one display with 6 memory items equally spaced and spanning 180 degrees, while another display with 2 groups of 3 items that are 180 degrees apart on the color space. To address these issues, the model we propose based on TCC suggests that the gist is simply a “summation” of the memory signals elicited by each item, as proposed in modeling how people make ensemble judgments (Robinson & Brady, 2023). We then suggested that different chunk-based factors can be read out of this summed representation. Our proposed model allows us to estimate the number, strengths, and positions of chunks in feature space, and which is not affected by outliers. We find that memory performance is better when the display has fewer chunks, when the overall gist is stronger, and when probed items are closer to the peak of gist.

### Set Size 6 Is Not Really Set Size 6?

If people exploit inter-item relationships when they try to hold the display in mind, counting the number of items is not sufficient to say how many “units” will be held in memory. At the extreme, it is unclear whether we should treat 6 items with exactly the same color hue as a single unit (e.g., Morey et al., 2015) or 6 units, or somewhere in between. We found that the numbers of chunks strongly affect memory performance, suggesting displays of 6 or 8 items may only rarely be truly “set size 6” or “set size 8” but may vary in their effective number of items depending on the exact structure of the display and its chunkability (similarly to models proposed by, e.g., Brady & Alvarez, 2015a; Brady & Tenenbaum, 2013).

How does the number of chunks affect the cost of adding more items (higher set size)? For example, it may not matter whether there are 2, 4, 6, or 8 items on the screen as long as they can be grouped into a single chunk. We can examine this in the data we analyze here (Figure 19). We find that the most chunkable color displays at set size 8 showing performance nearly as good as set size 3 displays, but the least chunkable showing much worse performance (Figure 19). This suggests that the real unit of analysis is not a “number of items” shown to the participant but critically depends upon the number of chunks present, even in randomly generated displays. Qualitatively, it also appears the chunks have a greater influence on color than orientation, though this may be an artifact of the chunking model being a better fit for color than orientation data (in particular, it does not consider location, and thus cannot model co-linearities and other effects that likely occur in orientation).

**Figure 18.**
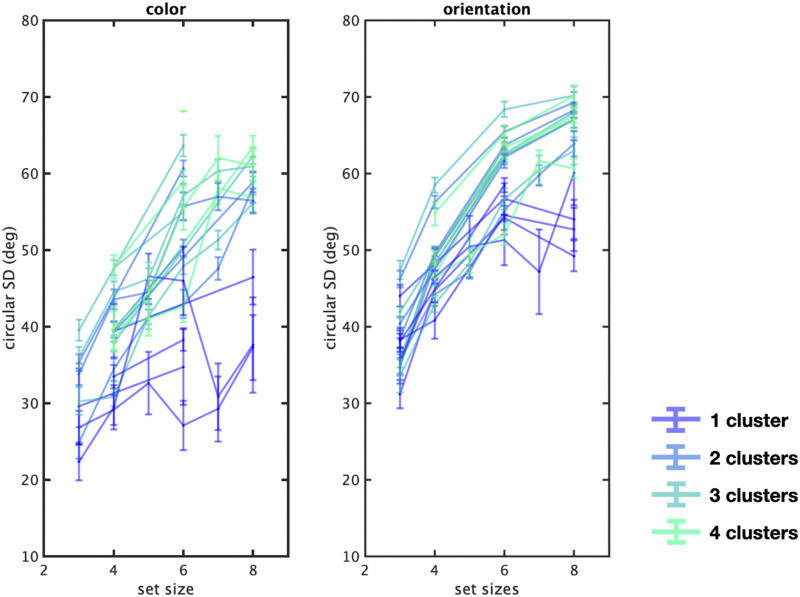
The chunkability of displays modulates performance across set size. The *y*-axis shows a measure of performance, the circular standard deviation, where higher numbers mean worse performance. More chunkable (blue/purple) displays result in much lower error, particularly at high set sizes, whereas less chunkable displays show a stronger set size effect (green/blue). Each line is a separate experiment.

These findings call into question many aspects of previous visual working memory models. Nearly all models in the literature assume all representations are based on independent items (see Bays et al., 2024 for a review), and here we show that these models do not capture major aspects of the patterns of responses in visual working memory experiments. Many debates in the visual working memory literature are based on the precise fit of extremely similar models that make nearly identical predictions except for slight details (see Bays et al., 2024 for a review), and our work suggests that these model-fit debates have been incorrect about a major aspect of how displays are encoded, calling all of these conclusions into question. This is especially true for debates about how performance changes with set size, which tends to be a major point of disagreement in this literature (e.g., the rate at which long-tailed errors arise is the core of slots+averaging models; Zhang & Luck, 2008). We show that chunking and grouping change systematically with set size with major implications for performance at the highest set sizes in particular (Figure 18), which is likely to affect all of the conclusions from model comparisons that are based solely on models that assume all representations are item-based even at higher set sizes.

### Attraction and Repulsion Between Chunks

In addition to effects of overall error being lower in more chunkable displays, we also found reliable effects of attraction and repulsion within and between chunks. These effects were also ubiquitous, and are not possible for individual item models to explain, as the combination of attraction and repulsion within the same displays makes clear that the ‘units’ of representation are not individual items.

We propose that these effects can be understood in much the same way previous works have explained why and when two items are attracted and repelled to each other (e.g., Bae & Luck, 2017; Chunharas et al., 2022; Golomb, 2015). In Chunharas et al. (2022), the experimenters showed that two items repelled from one another only when two items were (a U-shaped curve). When the experimenters made it more challenging to maintain two distinct memories such as by increasing the delay interval, this repulsion bias grew. But when two similar-colored items were added to the display, making 4 total similar items, the items became attracted toward each other rather than repelling.

In contrast to previous studies where the items were almost always found to be attracted toward the gist (e.g., Brady & Alvarez, 2011), here we found that analyzing the displays more carefully in terms of the particular chunks present—rather than just an overall gist—revealed that the direction of the biases depends on the level of distinctiveness between the items and the gist. Specifically, the items that are similar to the gist get attracted to the gist, and the ones that are dissimilar to the gist get repelled from the gist. This may have implications for real-world scenarios where both summarizing (attraction bias) and highlighting (discriminating, repulsion bias) might need to happen at the same time.

When participants have to remember a large number of items, one possibility is that they developed a gist-level representation which was quick and stable but less detailed, as well as item-level representations which are more detailed but take time to develop and are more challenging to maintain. In the current model, we define the gist color as the color at the peak of the sum of similarity functions (a kind of location-invariant sense of what colors were on the display). In thinking about which items are part of which chunk, the model associated each item with fuzzy feature estimations (each color is classified as the same or different group as the gist color).

**Figure 19.**
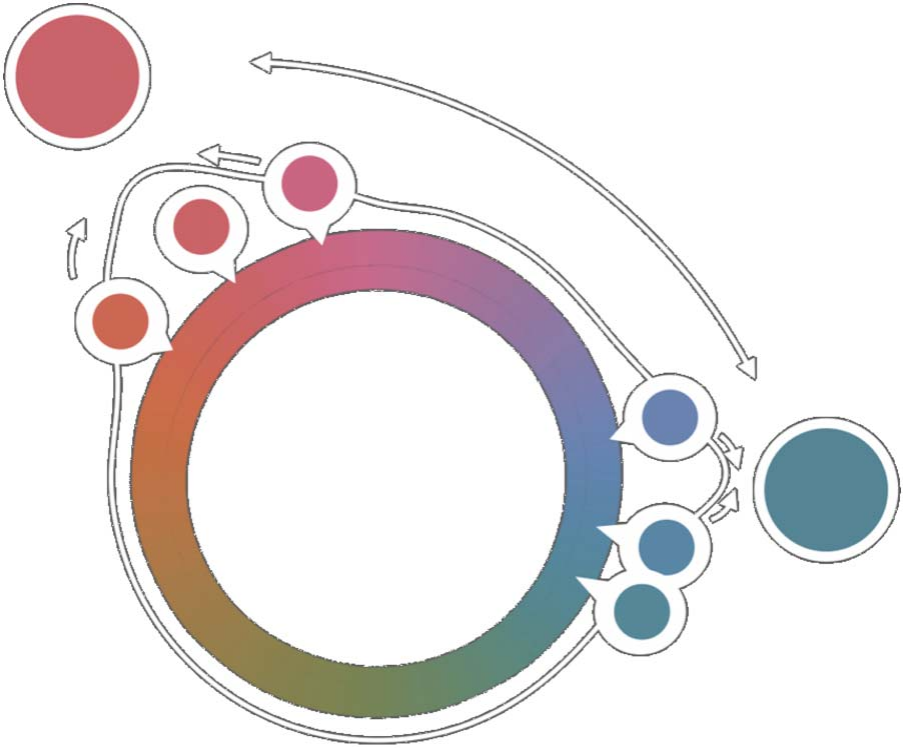
Schematic of what occurs on a display with two separate chunks, according to our data. Items within each chunk are attracted to the central gist color of that chunk. However, the two chunks also repel each other. In other words, people remember the chunks as more distinctive then they really were, but the items within them as less distinctive. This is similar to proposal about what people do with 2 vs. 4 item displays (e.g., Chunharas et al., 2022).

Importantly, when participants retrieved or responded to any of the targets, for example at set size 6 in the Adam et al. (2017) data, the data show that they not only used information about the current target but also utilized the information that they had about other items (i.e., “I might not remember the color of this particular target very well but I do remember that it was for sure not the (gist) color that I remember”; resulting in repulsion between the last reports and the first ones). A model that captures the general trend we observe in the data is shown in Figure 19; items within a chunk attract to that chunk’s central color/gist, whereas chunks themselves repel each other. Whether these processes happen during memory retrieval or at response/decision stages remains unclear, though Chunharas et al. (2022) argued that for set size 2 displays, they are not straightforwardly understandable by decision processes and seem to reflect genuine representational change.

### Set Sizes in the Real World

It is unclear why we would attempt to predict performance solely based on the number of simple items on a screen (e.g., ‘set size’), when in real-world settings defining the number of objects is challenging at best. In the real world, people encode scene gist and related global summaries of a scene (Manassi et al., 2017; Oliva, 2005) and use this to help constrain the individual items they remember (e.g., Lampinen et al., 2001), and even defining what counts as an item (A forest? A tree? A leaf?) can be quite challenging.

The discordance between counting the number of items on a simple segmented display and the make up of objects in real-world resonates strongly with the findings and debates in visual search studies, where again, scene gist strongly influences how many items are truly considered (e.g., Torralba et al., 2006). One of the classic findings is that people are slower at searching for a target with a higher “set size”. However, Wolfe et al. (2011) found that the search in real scenes is more efficient than what is predicted by set size manipulations, most likely because of the relatively higher predictability or compressibility of the real-world scene means defining an item that “counts” as part of the set size is impossible (Wolfe et al., 2011). Similarly, we suggest that even in the very simplest possible displays, designed to remove all structure, the true “set size” is not possible to compute, and does not reflect the number of segmented items shown but instead some internal processes of chunking and ensemble representations.

### Similarities and Differences From Previous Models

Our model overlaps to some extent with previous ideas and data about chunking and when it occurs and improves performance (e.g., Brady & Alvarez, 2015a; Son et al., 2020). For example, Brady and Alvarez (2015a) showed that simple color dot displays can be understood to some extent using a hierarchical model, where people cluster the items probabilistically into a cluster vs. outliers and then items within the cluster attract toward the mean. Our account also differs from existing accounts in its approach. For example, Orhan et al. (2014) showed that people are able to perform probabilistic inference by making use of statistical regularities to compress information from a working memory display (Orhan et al., 2014)—e.g., to represent it more efficiently than would be expected by the set size—especially when the regularities matched with natural statistics; similar results occur when people learn statistical regularities as well (e.g., Brady et al., 2009). This work suggests the limitations of visual working memory depend on both biological limitations and the ability to learn statistical regularities and make use of the structure of the world (see also Bays et al., 2024). Nassar et al. (2018) proposed that people are flexibly able to learn to optimally chunk the information (by shifting a threshold of how similar two items are to be treated as the same chunk) in order to reduce errors (Nassar et al., 2018). By contrast, our model makes a priori predictions about the locations and strengths of the gist(s) by summing perceptual similarity functions, rather than focusing on learning.

Our approach can, however, straightforwardly be extended to incorporate aspects of these previous ideas. For example, it is possible to examine how perceptual similarity functions change when people learn to discriminate between two or more categories, a kind of statistical learning that would be expected to change the similarity structure and thus the kinds of displays that allow chunking. Perceptual learning can lead to expanded representations in relevant feature dimensions (Goldstone, 1994), and category learning can warp psychological similarity space (Livingston et al., 1998; Nosofsky, 1986). For example, learning to distinguish between categories of colors (such as learning new color terms) may not improve the precision of early visual processing per se, but it does change how those colors are represented and compared in memory, leading to systematic changes in memory errors and confusability patterns (Roberson et al., 2005; Winawer et al., 2007). Thus, as similarity functions change with learning, this change in learned representational structure could affect which items will be treated as similar enough to be chunked together in working memory in our modeling framework.

### The Effects of Spatial and Feature Similarities

Our model only considers the summation of signals in feature similarity space without regard to where on the display they are located. Obviously this is a simplification, and location must matter as well to the chunks people form (as formalized by e.g., Brady & Tenenbaum, 2013).

One might ask whether our observed effects could be explained by existing item-based interference models, such as the one proposed by Oberauer and Lin (2017), that take into account item’s location. Their model accounts for reproduction errors in working memory through interference between items that overlap in both feature and context (e.g., location) representations. While this model can account for increased performance when items are similar in both feature and context, it does not predict other key phenomena we observe: (1) attraction to a global gist representation even when it is far from the individual item; (2) repulsion between chunks, which cannot be explained by any version of similarity-weighted blending from an item-based model; and (3) strategic response behaviors such as referring to the gist in output order.

Importantly, many datasets we analyze do not contain location information, precluding any direction evaluation of location-sensitive interference mechanisms (like that proposed by Oberauer & Lin, 2017) in this paper. However, unlike item-based models, our framework, grounded in chunk-level representations and gist-based integration, parsimoniously explains both attraction and repulsion patterns, including when similarity is low or gist is distant, and when participants strategically order their responses based on structured internal representations. Nonetheless, extending this framework to take into account location, as the Oberauer and Lin (2017) and other results (e.g., Brady & Tenenbaum, 2013) will clearly be critical to understand memory in its entirety.

Importantly, the approach we take here with summed similarity can be extended to include other robust grouping cues, especially spatial proximity, by adding the location similarity structure in much the same way as the color similarity structure, in a 2D representation of the display (similar to the way location and color are represented by e.g., Oberauer & Lin, 2017). Spatial-based and feature-based cues may also might also interact, with similarity in one dimension and the other being superadditive for chunking, given that a form of statistical regularity that is frequently encountered in nature is spatial regularities—things closer in space are usually more similar. However, this remains to be tested by future work.

## CONCLUSION

We investigated inter-item systematic biases in visual working memory using a variety of datasets. We found evidence for ubiquitous clustering, chunking, and non-item-based representations in working memory, rather than the traditional view of independent encoding of items. We propose that the gist in working memory is simply a “summation” of the memory signals, and that memory performance is better when displays have fewer gists, stronger gists, and probed items are closer to the peak of the gist. Hence, using set size alone as a measure of working memory capacity without considering inter-item relationships is far from complete. We also found that the items that were similar to the gist would be reported as even more similar to the gist while the ones that were dissimilar to the gist would be reported as even more dissimilar to the gist. These attraction and repulsion biases might reflect complex real-world scenarios where summarizing and highlighting may need to happen simultaneously.

## ACKNOWLEDGMENTS

We would like to thank Adam et al., Wilken & Ma, Zhang & Luck, Bays et al., Anderson et al., Anderson & Awh, van den Berg et al., and Rademaker et al., for making their data publicly available.

## FUNDING INFORMATION

This work was supported by NSF BCS-2146988 and the Exchange Faculty Travel Grant, Ratchadapisek Research Funds (#CTG168019) awarded to T.F.B. Support for C.C. was provided by PMU-B (#B44G660093) and the Faculty of Medicine, Chulalongkorn University, through the Ratchadapisek Research Matching Funds (#RA-MF-18/68).

## DATA AVAILABILITY STATEMENT

The data are publicly available on OSF at their original sources, or can be obtained from the corresponding author upon reasonable request, as cited in the manuscript.

## Figures and Tables

**Figure A1.**
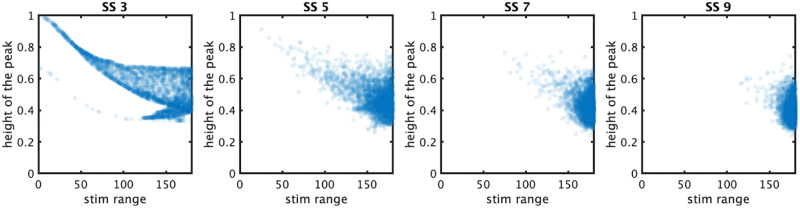
A simulation of relationship between the range of stimuli vs. height of the peak in the TCC chunking model at set sizes 3, 5, 7, 9. The range provides some information about the overall chunkability of the display (peak height in the TCC chunking model) at lower set sizes but this relationship falls apart at higher set sizes.

**Figure A2.**
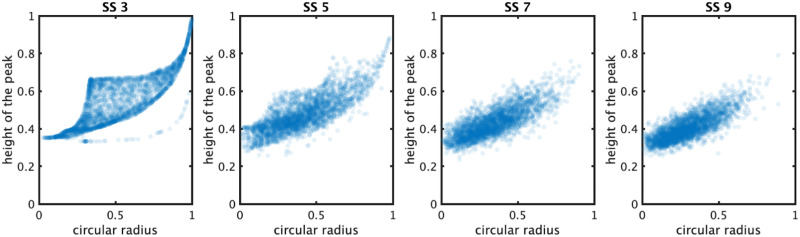
Simulation of relationship between circular resultant vector length vs. height of the peak in the TCC chunking model at set sizes 3, 5, 7, 9. The resultant vector length provides some information about the overall chunkability of the display (height of peak in the TCC-chunking model), particularly at higher set sizes.

**Figure A3.**
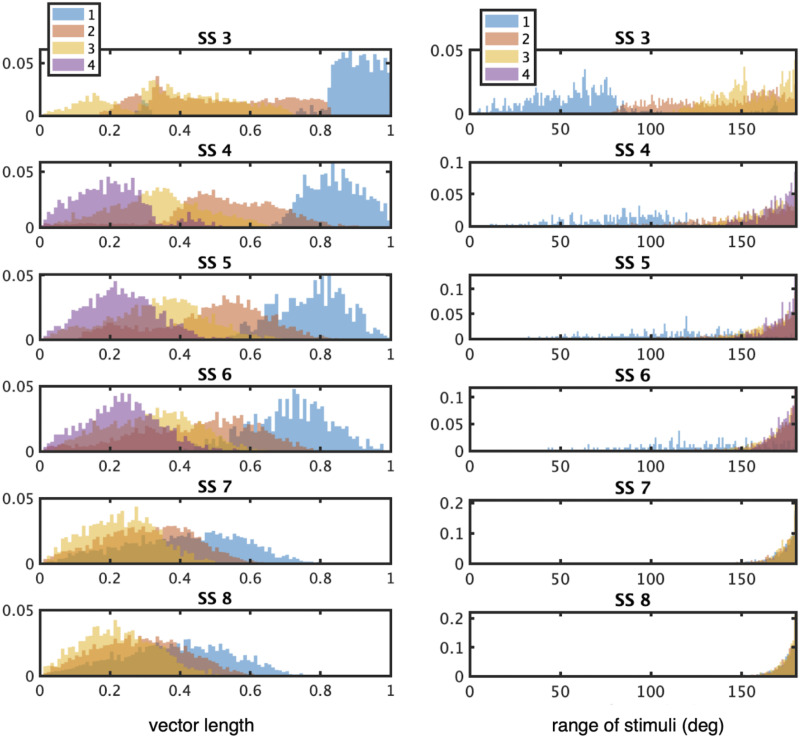
Mean resultant vector length (*x*-axis; left) and range of stimuli (*x*-axis; right) vs. number of chunks in the TCC-chunking model (1–4 chunks) at set sizes 3–8. Differences in resultant vector length provide information about how many chunks are present in the TCC model at low set sizes, but this falls apart at higher set sizes. The range provides information about number of chunks only at set size 3, for the most part, and not higher set sizes.

**Figure A4.**
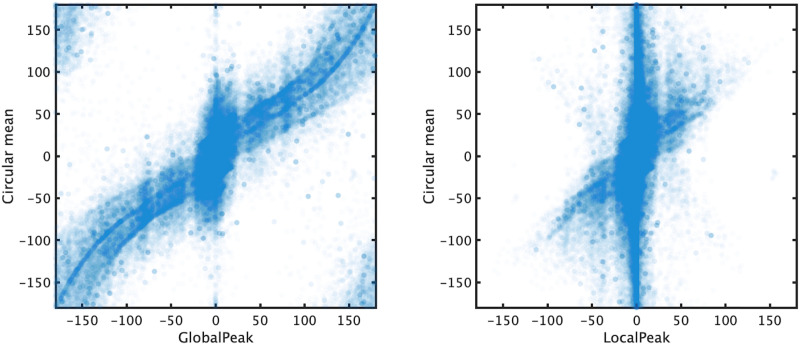
Relationship between the circular mean (*y*-axis) and the global gist location in the TCC chunking model (global peak: *x*-axis; left) and the local peak in the TCC chunking model (*x*-axis, right). The circular mean provides a noisy but approximate measure of the global peak, but insofar as we find most biases are best explained by attraction to local peaks and chunking based on local peaks, the circular mean provides basically no information about such peaks in the display.

**Figure A5.**
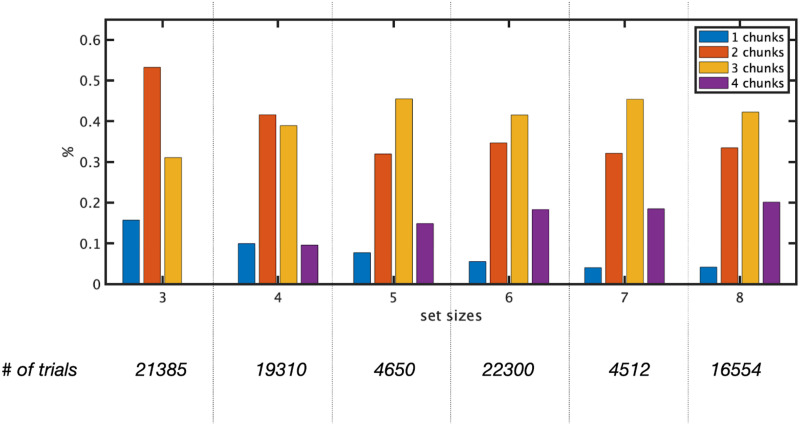
Total number of trials at each set size, and the proportion of them that contain each number of chunks. (Max. 4). We only show up to *N* = 4 chunks because there are almost never any displays with more than 4 chunks as we define them, given the width of the similarity function in these feature spaces. Each item causes activation that spreads over ±90 deg, with major activation up to ±45 deg at least, so items would have to be nearly perfectly spaced out equally, rather than randomly chosen, to have more than 4 chunks even at higher set size.

**Figure A6.**
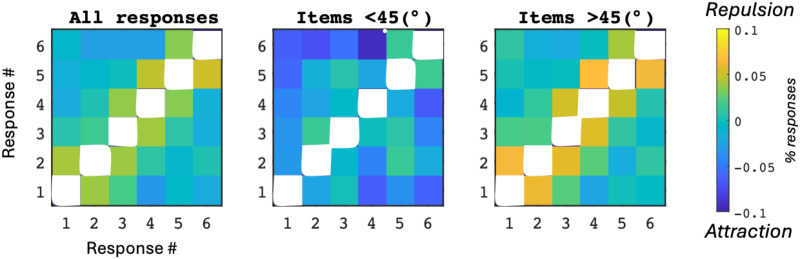
Considering all response pairs (response *N* vs. response *N* − 1 etc) we see repulsion effects between subsequent responses. However, this is largely driven by items that are far apart (e.g., where the item probed on response *N* is quite different than the item probed on response *N* − 1).

## APPENDIX

### Relationship Between the Circular Mean and Resultant Vector Length and the TCC-Based Chunking Model

Why has previous work not always found chunking effects, including a study that used the exact same datasets (van den Berg et al., 2014)? Previous work, with a few exceptions (e.g., Utochkin & Brady, 2020) has used measures that do not capture the structure of the displays in the way our chunkabillity model based on the TCC similarity function does. For example, van den Berg et al. (2014) focus on simply whether items are reported closer to the circular mean of all items in the display across all trials, with no attempt to select displays thatwho have a mean color that is possible to perceive. Other work has looked at the range of items on the display or the length of the circular mean vector (Brady & Alvarez, 2015a; Utochkin & Brady, 2020).

Here, we show how those relate to our chunking model. In particular, in simulations where we generate many displays with randomly chosen stimuli, mimicking the experimental stimuli analyzed in the main text, we find that while the range of items (e.g., the portion of the stimulus wheel they cover) provides a reasonable proxy for chunkability at set size 3, but it fails completely above that (Figure A1). The length of the mean vector captures some aspects of how chunkable the display is, particularly capturing the overall peak height (chunkability of the display) at higher set sizes (Figure A2), and providing information about how many chunks are present (Figure A3), but the circular mean is a very poor proxy for where this peak is located (Figure A4).

### Number of Chunks

In the main text, we analyze performance as a function of the number of chunks present in a display according to the TCC-chunking model. Figure A5 shows a count of how often each number of chunks occurs at each set size.

### Relationship Between Subsequent Responses

In the main text, we analyze how responses to item *N* in the Adam et al. (2017) dataset relate to the stimulus *N* − 1, *N* − 2 etc. That is, we focus on how responses relate to stimuli.

An alternative question is about how responses relate to other responses (e.g., a kind of response-based serial dependence). To examine this, we can look at how response *N* relates to response *N* − 1. We do so in the Adam et al. (2017) forced-response (random probe) experiments. We focus on the random probe conditions of Adam et al., because this means that previous stimuli are controlled, which seems to be the appropriate way to understand response effects, as it is independent of people’s propensity to report items in chunk-based orders, which we find in the free report conditions.

Do we find response-based serial dependence, where responses to item *N* are attracted to responses to item *N* − 1? No. We do not find any main effect of items being attracted to previous responses. We find a small effect in the opposite direction. That is, if you compare report *N* to report *N* − 1 (not to stimulus *N* − 1, as we do in the main paper), you find that on average people report item *N* as further away from *N* − 1 than would be predicted based on the item they saw (*t*(16) = 2.85, *p* = 0.012). The further away responses, that is, responses that are not subsequent but differ by 2 or more response, show no attraction or repulsion (*t*(16) = 1.31, *p* = 0.210).

When we look only at pairs of reports where the stimuli being probed at time *N* are somewhat similar (within 45 deg on the wheel) to the stimuli being probed at time *N* − 1, we find no reliable attraction or repulsion. Instead, the repulsion largely comes from subsequent responses to stimuli that are far apart on the wheel. In particular, a 2 × 2 ANOVA, with stimulus distance as on factor and subsequent vs. non-subsequent responses as the other factor, shows a reliable interaction, driven by more repulsion for subsequent reports for far away items (*F*(1, 16) = 6.3772, *p* = 0.02). This seems most consistent with an effect of meta-knowledge of some kind, e.g., people’s knowledge about whether items were or were not part of the same cluster, where they tend to want to report something different on trial *N* then *N* − 1 if they know the items were different.
